# Ventilator-induced lung injury results in oxidative stress response and mitochondrial swelling in a mouse model

**DOI:** 10.1186/s42826-022-00133-4

**Published:** 2022-07-22

**Authors:** Jon Petur Joelsson, Arni Asbjarnarson, Snaevar Sigurdsson, Jennifer Kricker, Bryndis Valdimarsdottir, Holmfridur Thorarinsdottir, Eir Starradottir, Thorarinn Gudjonsson, Saevar Ingthorsson, Sigurbergur Karason

**Affiliations:** 1 University of Iceland, Reykjavík, Iceland; 2 Landspitali University Hospital, Reykjavik, Iceland; 3 EpiEndo Pharmaceuticals, Reykjavik, Iceland

**Keywords:** Ventilator-induced lung injury, Oxidative stress, Mouse studies, Acute lung injury

## Abstract

**Background:**

Mechanical ventilation is a life-saving therapy for critically ill patients, providing rest to the respiratory muscles and facilitating gas exchange in the lungs. Ventilator-induced lung injury (VILI) is an unfortunate side effect of mechanical ventilation that may lead to serious consequences for the patient and increase mortality. The four main injury mechanisms associated with VILI are: baro/volutrauma caused by overstretching the lung tissues; atelectrauma, caused by repeated opening and closing of the alveoli resulting in shear stress; oxygen toxicity due to use of high ratio of oxygen in inspired air, causing formation of free radicals; and biotrauma, the resulting biological response to tissue injury, that leads to a cascade of events due to excessive inflammatory reactions and may cause multi-organ failure. An often-overlooked part of the inflammatory reaction is oxidative stress. In this research, a mouse model of VILI was set up with three tidal volume settings (10, 20 and 30 mL/kg) at atmospheric oxygen level. Airway pressures and heart rate were monitored and bronchoalveolar lavage fluid (BALF) and lung tissue samples were taken.

**Results:**

We show a correlation between increased inflammation and barrier failure, and higher tidal volumes, evidenced by increased IL-6 expression, high concentration of proteins in BALF along with changes in expression of adhesion molecules. Furthermore, swelling of mitochondria in alveolar type II cells was seen indicating their dysfunction and senescence-like state. RNA sequencing data present clear increases in inflammation, mitochondrial biogenesis and oxidative stress as tidal volume is increased, supported by degradation of Keap1, a redox-regulated substrate adaptor protein.

**Conclusions:**

Oxidative stress seems to be a more prominent mechanism of VILI than previously considered, indicating that possible treatment methods against VILI might be identified by impeding oxidative pathways.

**Supplementary Information:**

The online version contains supplementary material available at 10.1186/s42826-022-00133-4.

## Background

Mechanical ventilation (MV) is a lifesaving feature in the treatment of critically ill patients. The purpose of MV is to ensure sufficient gas exchange while taking over the work of breathing during the resolution of the underlying disease that caused the respiratory failure. Unfortunately, MV can lead to excessive mechanical strain to lung tissues causing damage to them, especially in patients already suffering lung diseases. This has been named ventilator-induced lung injury (VILI) [1].

The difference between normal spontaneous breathing and mechanical ventilation underlines the phenomena of VILI. Normal respiration relies on generating negative pressure in the lungs, causing air to flow towards low pressure. This negative pressure is generated mostly by contraction of the intercostal muscles and diaphragm. Relaxing these muscles creates positive pressure in the lungs and air is expired. During MV, air is thrust with positive pressure into the patients’ lungs for efficient gas exchange at the possible risk of damaging the lung epithelium due to mechanical strain [2].

The four main hallmarks of VILI have traditionally been baro/volutrauma, atelectrauma, oxygen toxicity and biotrauma [3]. Baro/volutrauma occurs when using inappropriately high airway pressures or tidal volumes subjecting open alveoli to over-distension. Atelectrauma follows the shear stress of tissues caused by repeated opening and closing of the alveoli when the positive end-expiratory pressure (PEEP) setting in the ventilator is too low. The use of supraphysiological levels of oxygen may cause toxicity in tissues by interrupting cellular processes due to formation of reactive oxygen species [4]. Biotrauma is the eventual activation of immune cells because of the aforementioned injuries to the tissues, which results in a cascade of effects from an over-stimulated immune response leading to excessive circulating inflammatory markers that can affect other organs, leading to organ failure in the most critical cases [5].

A possibly underappreciated mechanism of VILI is excessive oxidative stress following the inflammatory reaction, causing a further imbalance in favor of the oxidants [6]. Through the generation of reactive oxygen species and peroxides, this disturbance can interrupt the cells’ ability to detoxify reactive species and repair the ensuing damage. Redox reactions are therefore very important regulators of the oxidation state of atoms and can occur at both the enzymatic and transcriptional levels [6].

Increased oxidative stress is implicated in numerous diseases, including lung diseases such as chronic obstructive pulmonary disease (COPD) [7–9] and acute respiratory distress syndrome (ARDS) [10]. Oxidative stress is also linked to cellular aging and senescence [11].

Nuclear factor-erythroid 2-related factor 2 (Nrf2 (*Nfe2l2*)) is the master regulator of anti-oxidant responses and is an important regulator for the resolution of inflammation [12]. As an anti-inflammatory mediator, Nrf2 has been shown to inhibit expression of pro-inflammatory cytokines, such as IL-1β, IL-6 and TNF-α [13]. Glutathione (GSH) is a potent antioxidant present in all mammalian tissues and acts as a key antioxidant in the body, defending against oxidative stress [14, 15]. Nrf2 is crucial for the production of GSH as it is the solitary regulator of the enzymes that facilitate its production [16]. Nrf2, under normal conditions, is rapidly degraded by Kelch-like ECH associating protein 1 (Keap1). The Nrf2-Keap1 complex is targeted by signals from, for example, reactive oxygen species, stabilizing Nrf2, which translocates to the nucleus and resumes its activities as a transcription factor [17].

Using a mouse model of VILI, we set out to investigate the relationship between VILI and oxidative stress, and the impact of oxidative stress on VILI-associated parameters. We show that higher tidal volumes decrease the epithelial barrier in the lungs, by way of increased protein concentration and IL-6 inflammatory marker production in BALF. Higher tidal volumes result in swelling of mitochondria and intensified oxidative stress on the transcriptional level, along with increased glutathione pathway expression.

## Results

### High tidal volume results in decreased barrier protection and increased inflammatory response

**Fig. 1:**
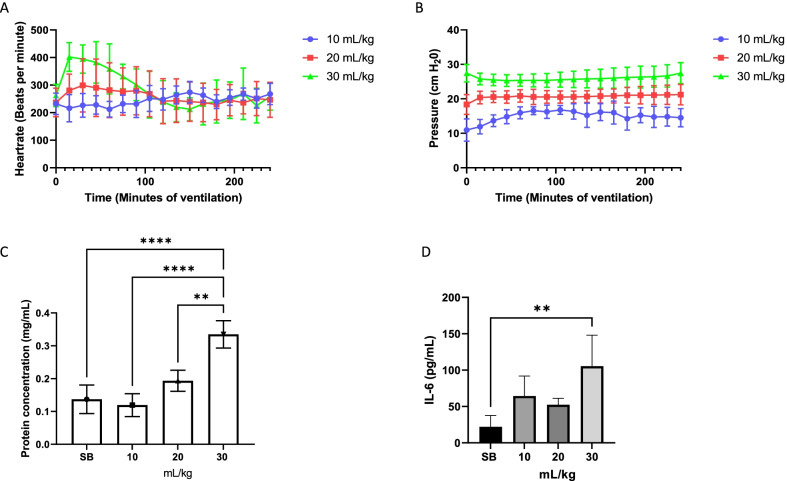
High tidal volume results in decreased barrier protection and increased inflammatory response. When ventilating the mice at different tidal volumes, heart rate remained stable for the two lower tidal volumes. However, in the initial first hour of ventilation with tidal volume of 30 mL/kg heart rate increased but then remained stable (**A**). Pressure needed to maintain the applied tidal volumes remained relatively stable throughout the ventilation period (**B**). Increased protein concentration in BALF was detected in the 30 mL/kg tidal volume range (**C**). Pro-inflammatory cytokine, IL-6, expression increased significantly in the mice that were ventilated at 30 mL/kg (**D**). Standard deviation of the means is shown. Significant difference from the SB controls are shown (*P* ≤ 0.01 = **; *P* ≤ 0.0001 = ****)

Three different groups of mice were ventilated with atmospheric air at 10, 20 and 30 mL/kg of tidal volume for four hours, during which heart rate was monitored to observe their vital signs. Interestingly, heart rate remained stable in the mice at both 10 and 20 mL/kg tidal volume, but initial heart rates increased in mice ventilated at 30 mL/kg then levelled off after two hours (Fig. 1A). Increased tidal volume caused increased airway pressures and required increased pressure output from the mechanical ventilator (Fig. 1B). Total protein concentration in the BALF was measured after ventilating the mice for 4 h. A significant increase in total protein concentration was evident in the mice ventilated at 30 mL/kg tidal volume (Fig. 1C). A similar pattern was seen when the pro-inflammatory marker, IL-6, was measured in the BALF (Fig. 1D).

### Increased tidal volume leads to decreased tissue area and a more open airway

**Fig. 2:**
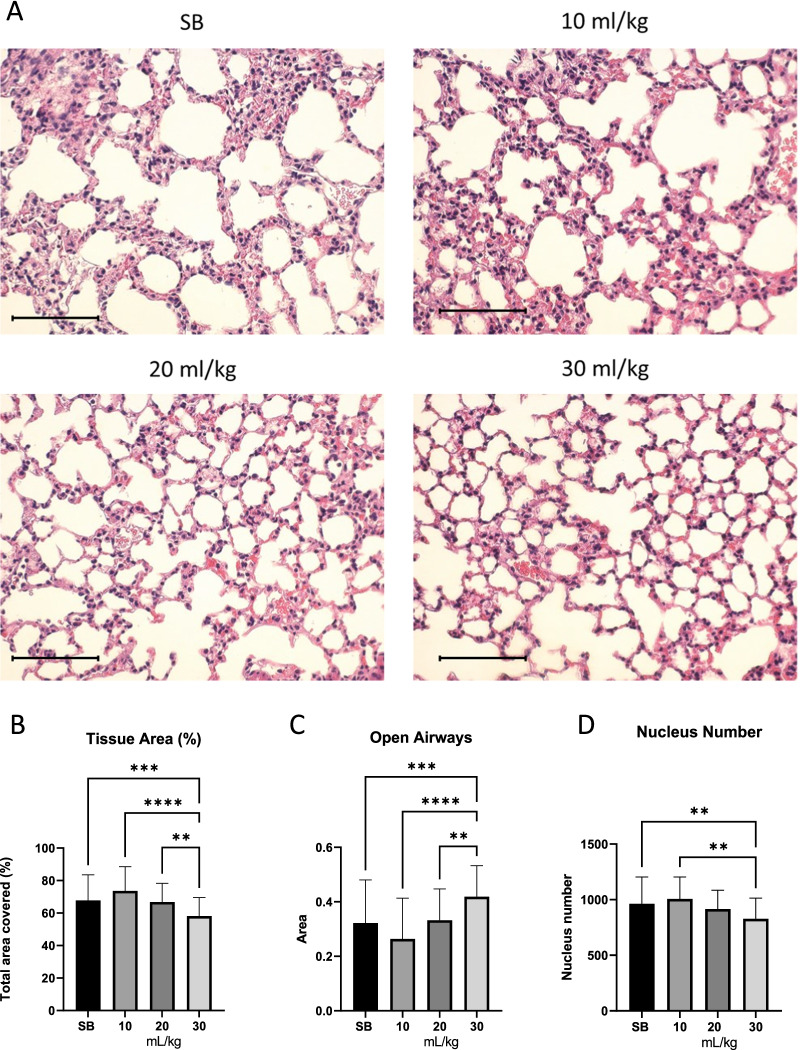
Tissue area decreases as tidal volume increases. Shown are representative images of lung alveoli in SB and ventilated mice (**A**). Tissue area decreased significantly in mice ventilated at 30 mL/kg (**B**), while open airways increased (**C**). Total nucleus number decreased in mice ventilated at 30 mL/kg (**D**). Standard deviation of the means is shown. Significant difference from the SB controls are shown (*P* ≤ 0.01 = **;* P* ≤ 0.001 = ***; *P* ≤ 0.0001 = ****). Scale bar is 100 µm

To study the effect of mechanical ventilation, tissue samples from ventilated mice were compared to those spontaneously breathing (SB), that were sacrificed immediately after tracheotomy, and were not mechanically ventilated. Figure 2A shows a comparison of representative images of lung tissue cross-sections. See Additional file 1: Figure S1 for whole lung scans. Image analysis of 6 different mouse lungs shows that the collapsed tissue area is considerably decreased in 30 mL/kg tidal volume ventilated mice in comparison to SB and lower tidal volumes (Fig. 2B). Airways are significantly more open in 30 mL/kg tidal volume ventilated lungs (Fig. 2C). The number of nuclei in 30 mL/kg tidal volume ventilated mice is significantly decreased when compared to SB and 10 mL (Fig. 2D), presumably in connection to the relatively more open airways.

### Increments in tidal volume lead to increase in significantly differentially expressed genes

To gain a good overview of events occurring during MV, lobes from lungs were utilized for RNA sequencing. Gene expression from mice ventilated with 10, 20 and 30 ml/kg tidal volumes was compared to SB mice.

**Fig. 3:**
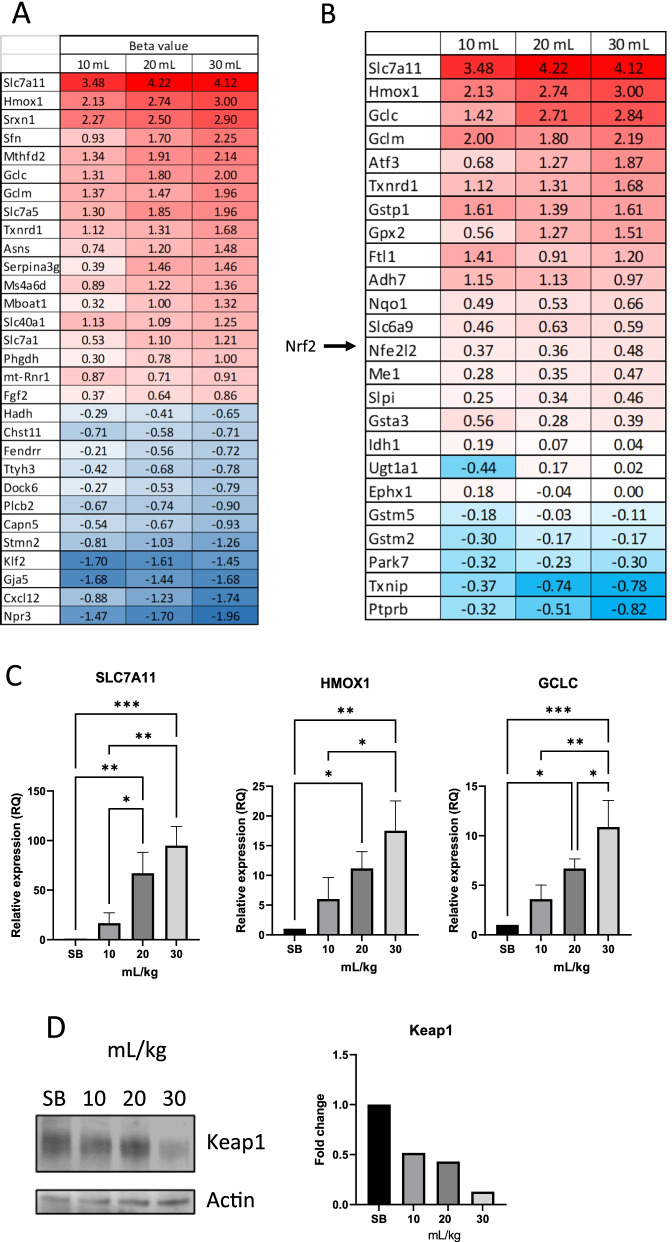
Top 30 differentially expressed genes. Increased tidal volume leads to increased differential expression. Shown are the top 30 genes with the highest beta value from RNA sequencing of mouse lung tissues. Up regulated genes showed increased expression with increased tidal volume, while down regulated genes decreased in expression with increased tidal volume (**A**). Genes involved in glutathione production were significantly upregulated. The expression was shown to be tidal volume dependent (**B**). Real time PCR was done using lung tissue from ventilated mice and compared to SB controls (**C**). The top three genes from sequencing analysis were confirmed to be expressed in a tidal volume dependent manner. Immunoblotting from SB control, 10, 20 and 30 mL/kg tidal volume ventilated mice was performed for Keap1 and Actin. Actin used as a loading control and quantification carried out using FIJI (**D**). Significant difference from the SB controls are shown (*P* ≤ 0.05 = *; *P* ≤ 0.01 = **;* P* ≤ 0.001 = ***)

As seen in Fig. 3A, in almost all cases a stepwise incremental increase/decrease, related to tidal volume, is seen in the top 30 significantly differentially expressed genes. A number of the top upregulated genes are involved in glutamate/glutathione metabolism.

### Differentially expressed genes associated with Nrf2 activation

Nrf2 (*Nfe2l2*) is a transcription factor and established master regulator of the anti-oxidative and detoxifying cellular response [18] and been shown to be protective against lung injury [16]. Nrf2 is normally bound by Keap1 [17]. Autophagosomal degradation of Keap1 leads to activation of Nrf2 and subsequent translocation into the nucleus [19–21]. Once inside the nucleus, Nrf2 induces the expression of important cytoprotective genes. These include genes responsible for detoxification, antioxidation and metabolism, along with inhibiting the expression of pro-inflammatory genes [12]. Nrf2 is a regulator of enzymes that facilitate the production of glutathione [16]. When looking for downstream effector genes of Nrf2 in our sequencing analysis, we saw a trend of increased differential expression with increased tidal volume (Fig. 3B). Expression of the top 3 most significantly over-expressed genes, *SLC7a11*, *HMOX1* and *GCLC*, were confirmed with real-time PCR (Fig. 3C). Western blots of lung tissue samples from the mice showed a clear breakdown of Keap1 (Fig. 3D).

### Gene set enrichment analysis of different tidal volume outputs

Gene expression was examined with relation to known pathways using gene set enrichment analysis (GSEA). GSEA shows the degree to which a set of genes is overrepresented at the top or bottom of a ranked list of genes [22, 23]. A positive enrichment score indicates enrichment of the gene set at the top of the ranked list, while a negative enrichment score reflects the enrichment of the gene set at the bottom of the ranked list. The primary statistic for examining gene set enrichment results is the normalized enrichment score (NES). NES accounts for differences in gene set size and in correlations between gene sets and the expression dataset.

**Fig. 4:**
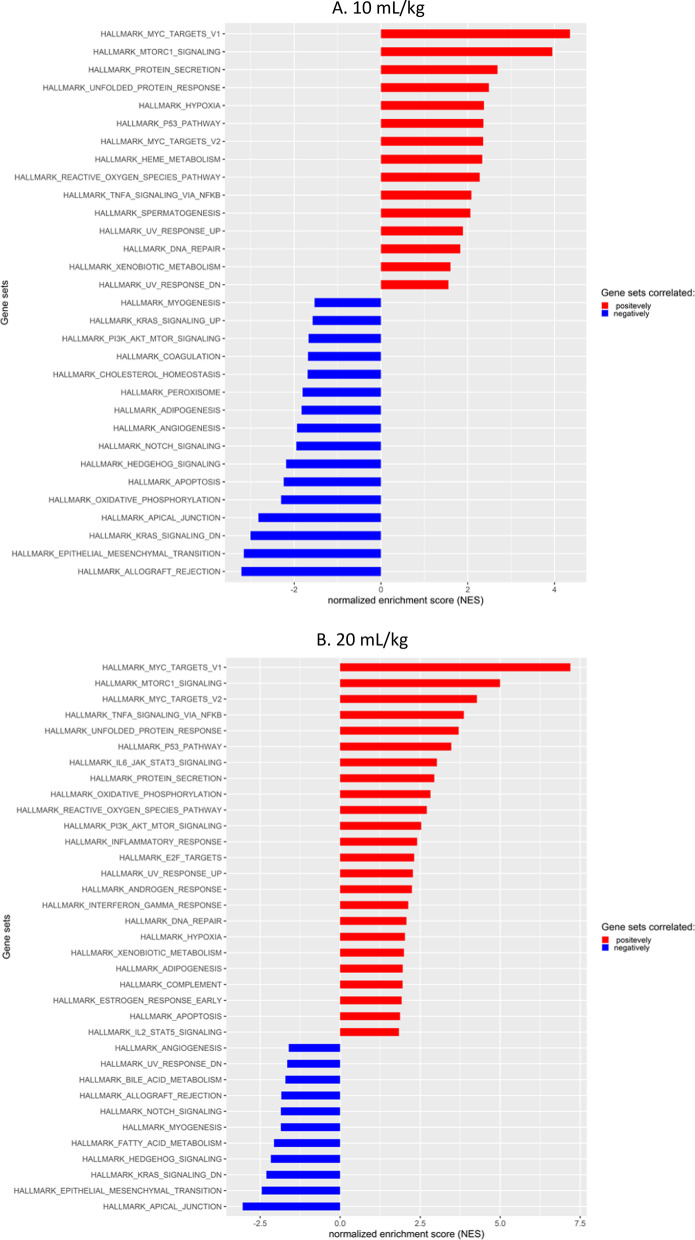

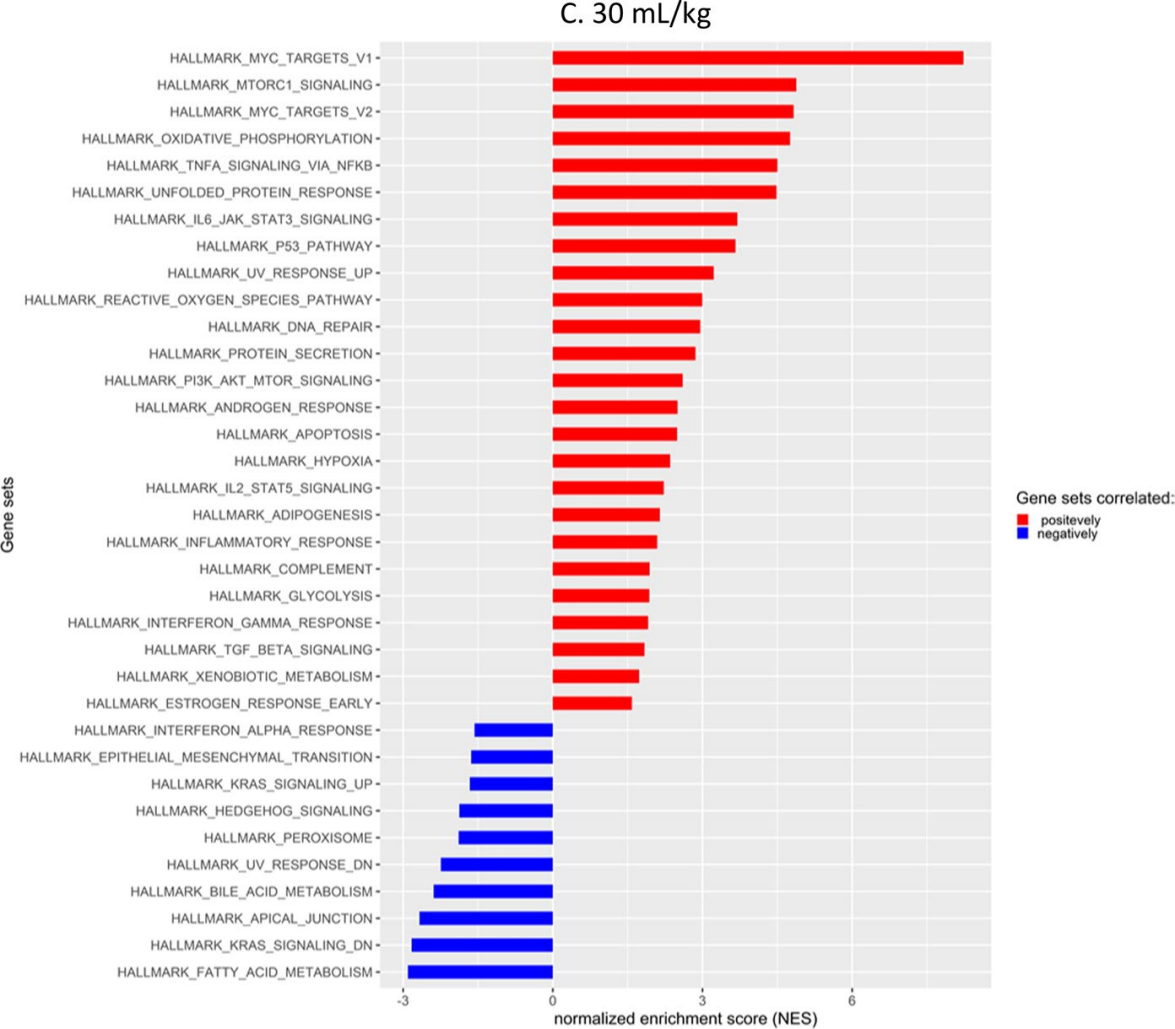
Gene set enrichment scores. Increased NES due to tidal volume increase. Gene set enrichment analysis from mouse tissue RNA sequencing, comparing SB to increased tidal volumes

Figure 4A–C shows a bird’s eye view of the NES of different pathways caused by different tidal volumes. Gene sets related to mitochondrial activity and oxidative stress were enriched. These include MYC_TARGETS_V1, MYC_TARGETS_V2, MTORC1_SIGNALING, OXIDATIVE_PHOSPHORYLATION and REACTIVE_OXYGEN_SPECIES [24–28]. Generally, the NES was increased stepwise correlating with an increase in tidal volume when viewing positive enrichment scores for these gene sets. Interestingly, genes associated with apical junctions were reduced, although real-time PCR of the tight junction gene, *Cldn4*, showed a stepwise increase in expression as tidal volume was increased (Additional file 2: Figure S2)

### Gene sets pertaining to mitochondrial biogenesis and respiration are enriched as a result of higher tidal volume

**Fig. 5:**
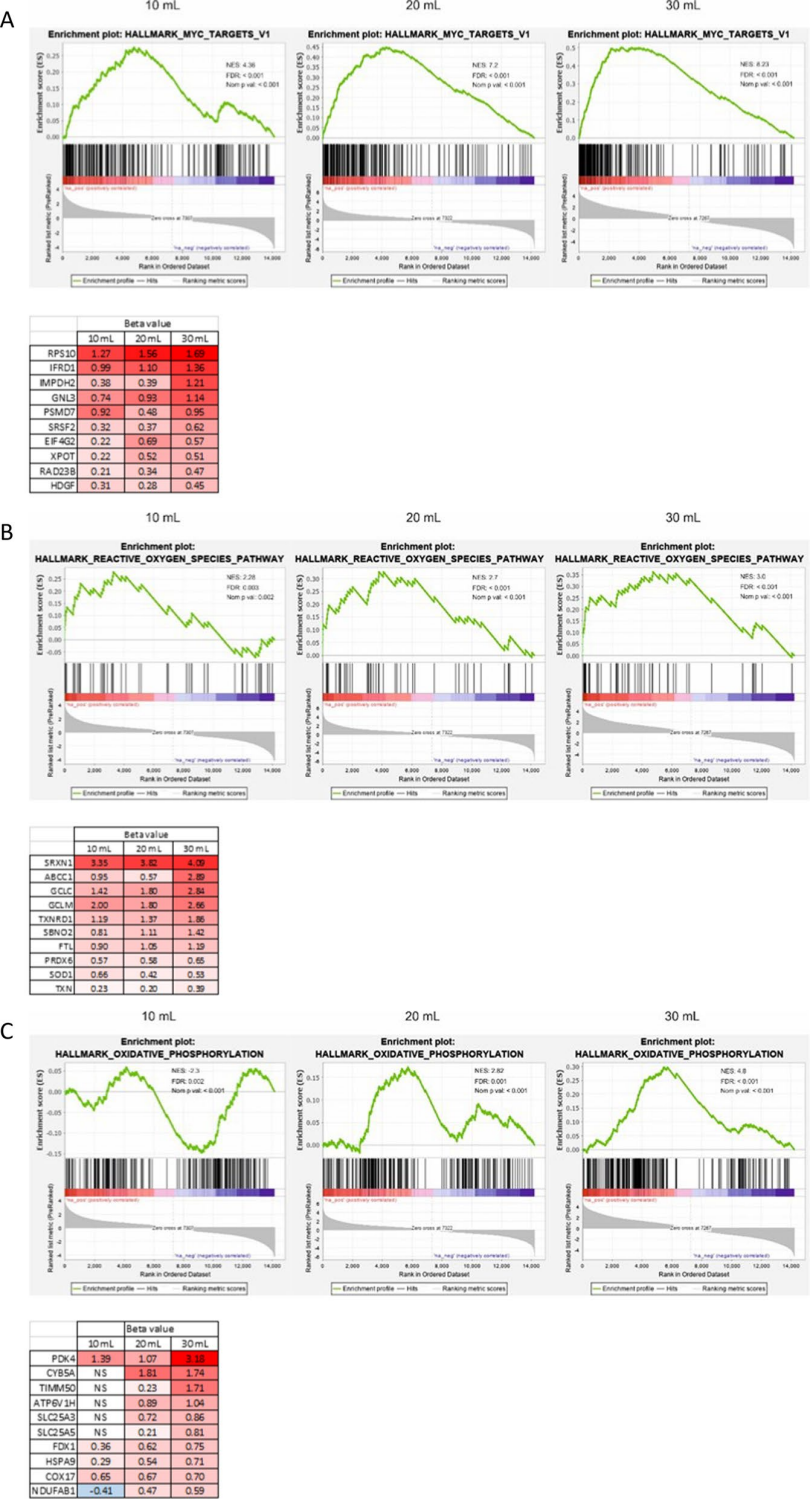

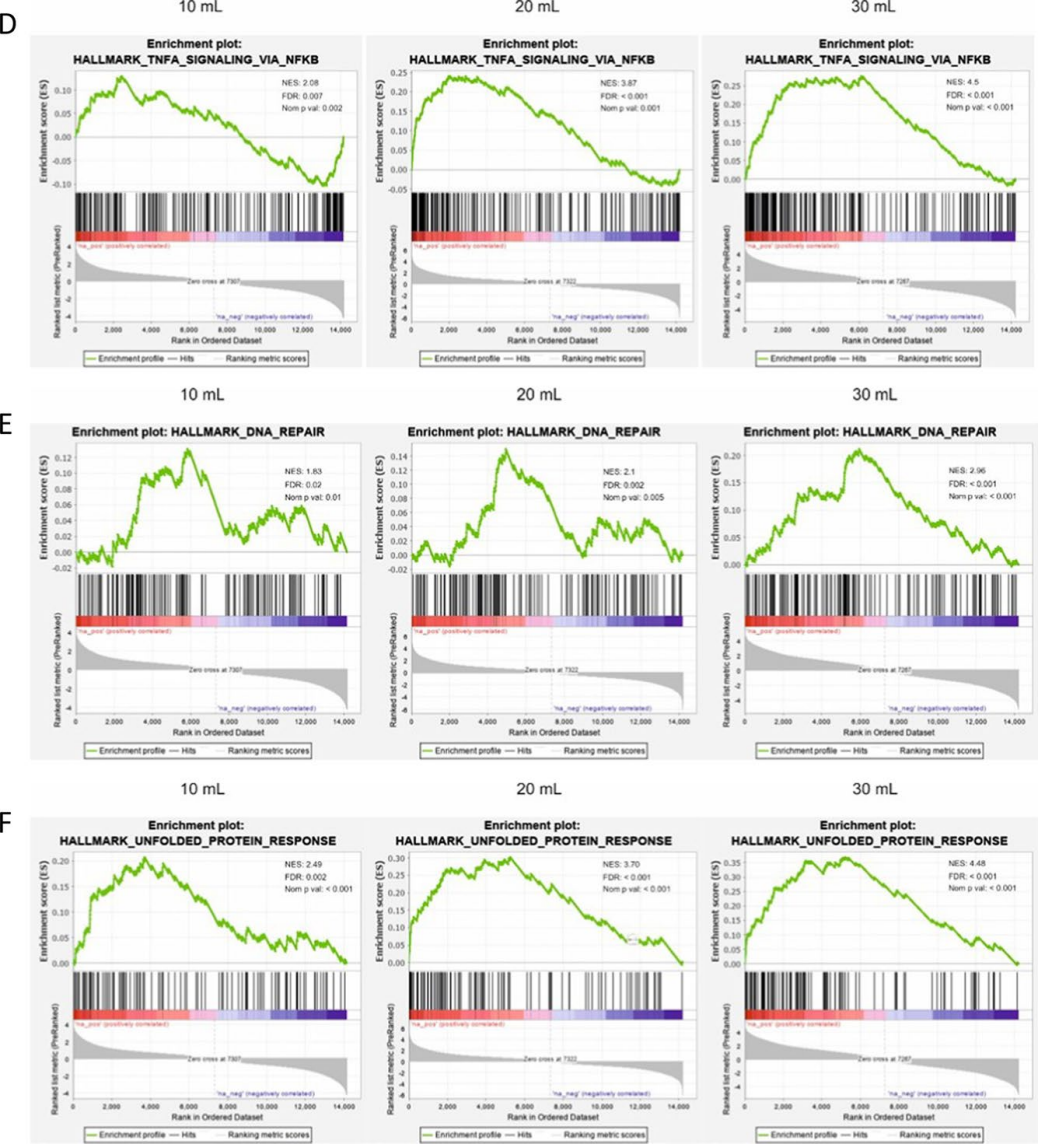
Genes set pertaining to mitochondrial biogenesis and respiration are enriched as a result of higher tidal volume. HALLMARK_MYC_TARGET_V1, REACTIVE_OXYGEN_SPECIES_PATHWAY and OXIDATIVE_PHOSPHORYLATION gene sets were enriched dependent on tidal volume increases (**A**–**C**). The top 10 genes with highest beta value from the appropriate gene sets are displayed beneath each graph. TNFA_SIGNALING_VIA_NFKB, DNA_REPAIR and UNFOLDED_PROTEIN_RESPONSE gene sets increase their NES scores with increased tidal volumes (**D**–**F**)

As oxidative stress-related gene sets were being enriched, we took a closer look at the gene sets and top genes responsible. The highest NES across all tidal volumes was seen in MYC_TARGETS_V1, namely, 4.36 at 10 mL/kg, 7.2 at 20 mL/kg to 8.26 at 30 mL/kg. Tabularizing the top differentially expressed genes, when comparing SB mice to mice that received 30 mL/kg tidal volume, revealed a positive correlation between gene expression and tidal volume (Fig. 5A). A similar trend was seen with both gene sets of REACTIVE_OXYGEN_SPECIES (NES increased from 2.28 to 3.0) (Fig. 5B) and OXIDATIVE_PHOSPHORYLATION (NES increased from neg (–) 2.3 to 4.8) (Fig. 5C). Displayed beneath each enrichment plot figure are the top 10 upregulated genes in the gene sets (Fig. 5A–C). Many of the top differentially expressed genes from these gene sets followed the same pattern of increasing expression. OXIDATIVE_PHOSPHORYLATION genes from the 10 mL/kg tidal volume mice were negatively correlated and accordingly, many of the genes were not significantly (NS) expressed (Fig. 5C). Importantly, other inflammation-related gene sets were also increased due to increased tidal volume, such as TNFA_SIGNALING_VIA_NFKB, DNA_REPAIR and UNFOLDED_PROTEIN_RESPONSE (Fig. 5D–F).

### Mitochondrial phenotypic changes

**Fig. 6:**
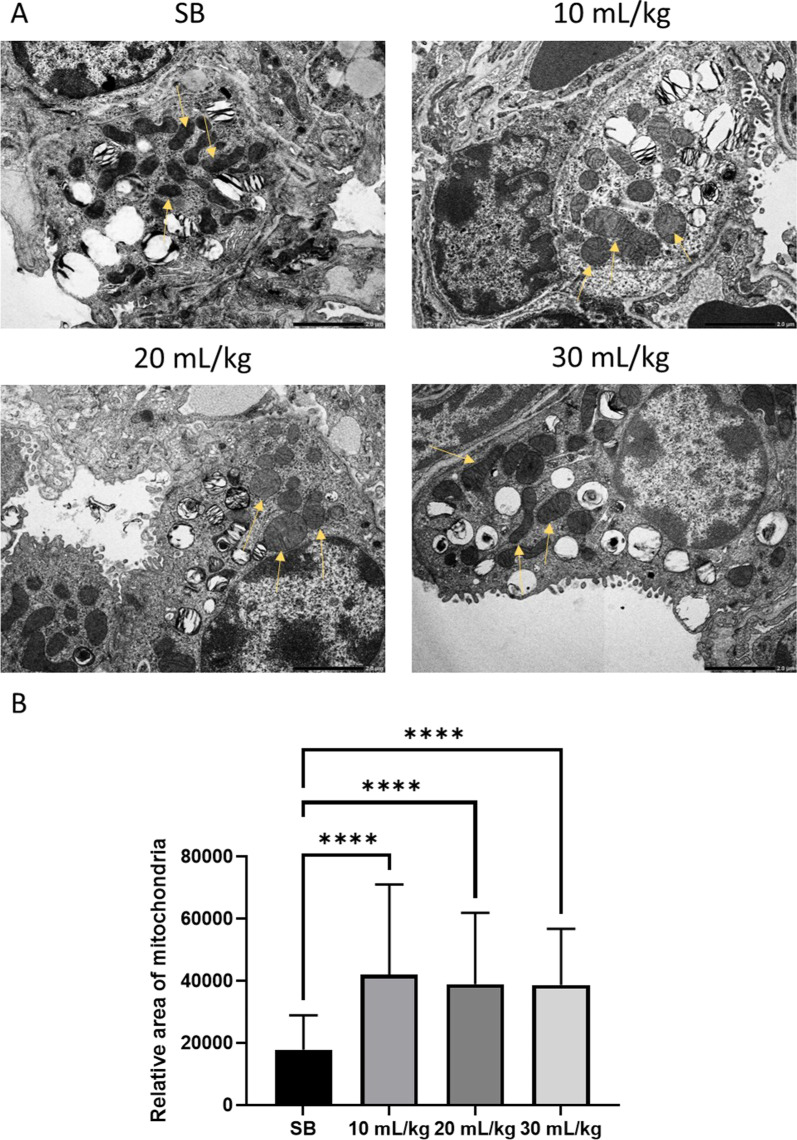
Increased area of mitochondria in ventilated mice. Mitochondrial swelling was observed in ventilated mice (**A**). Mitochondria were traced in images, using FIJI image processing (**B**). Mitochondria in ventilated mice were significantly larger. Standard deviation of the means is shown. A range of 41–84 measurements of mitochondria at the same magnifications were performed. (*P* ≤ 0.0001 = ****)

As mitochondrial swelling in alveolar type 2 (AT2) cells had been reported in ventilation studies [29], transmission electron microscopy (TEM) was utilized for a closer inspection of the mouse tissue after ventilation. As seen in Fig. 6, MV resulted in a significant increase in AT2 mitochondrial swelling in our model as well.

## Discussion

In this study, we have analyzed the extent of VILI in a mouse model using three different tidal volumes 10, 20 and 30 mL/kg and compared to spontaneously breathing mice. Possible augmentation of VILI by oxygen toxicity due to a high ratio of inspired oxygen was excluded by ventilation with atmospheric air. Total protein concentration and pro-inflammatory markers were increased in BALF indicating barrier failure and inflammation, respectively. Gene expression analysis demonstrated that expression of genes associated with oxidative stress was strongly enriched with higher tidal volumes. Associated with these observations, phenotypic changes in mitochondria were detected as evidenced by their enlargement at 10, 20 and 30 mL/kg tidal volume compared to SB mice. In addition, histological analysis showed reduced tissue area, increased open airspaces, and reduced number of nuclei in mice that were MV with 30 mL/kg compared to SB. This might appear as being counterintuitive, as one would beforehand predict inflammation and closing of the airways upon high levels of ventilation. Yet, this could be due to our method of fixing and staining the lung tissue. Most likely, the SB lungs collapsed extensively after anaesthesia, tracheostomy and euthanasia, as no opening of alveoli occurred after the tracheostomy nor stiffening caused by VILI had occurred. In the 10 mL/kg group the lungs have been partly reopened while in the 20 and 30 mL/kg ventilated mice, opening, eventual overstretching and stiffening of the tissue was evident. While physiological effects weren’t obvious, the effects can be seen in the histological analysis, as indicated by less tissue collapse, more open airways and fewer cells per field of view.

Mouse models of VILI have shown that overstretching the lung tissues increases the inflammatory response in mice along with damaging the lung epithelium [3]. Setting up our own mouse model of VILI, we set out to identify important genes involved in the transcriptional response to mechanical ventilation of different tidal volumes.

Tidal volumes were set according to mL/kg and as seen in Fig. 1, the pressure output of the ventilators was stable for each tidal volume setting. Heart rate was also stable across the two lower tidal volume settings while the initial heart rate increased substantially in the highest tidal volume group but lowered after about two hours of ventilation. The minute ventilation (breaths/minute * tidal volume) for the 20 and 30 mL/kg groups were set at 2400 mL. In the lowest tidal volume group, 10 mL/kg, we decided to decrease the minute ventilation to 1500 mL, as we deemed 240 breaths/minute too high and could lead to excessive mechanical strain compared to the other two. This might have led to higher CO_2_ levels in the lowest tidal volume group.

Protein concentration in the BALF was not changed at 10 mL/kg and only rose slightly at 20 mL/kg, while increasing almost threefold in the highest tidal volume, 30 mL/kg. This might indicate that a pressure level was reached where damage was occurring in the lung epithelium resulting in barrier failure and accumulation of proteins in BALF. Furthermore, increased concentration of the pro-inflammatory marker IL-6 was detected in the BALF, and this was significantly higher in the 30 mL/kg group, indicating that the 30 mL/kg was causing increased damage to the lungs. IL-6 has been shown to be upregulated during traumatic events in the respiratory tissue [30].

Histology analysis were not as anticipated, with higher tidal volume ventilation one would expect to see for example edema and hemorrhage [31]. We however report a more open histology as an effect of high ventilation, due to lungs being fixed in formalin without inflating during fixing [32]. Resulting in histology of the SB control being more closed due to the tissue being uncompromised by ventilation. The ventilated samples however, due to baro/volutrauma have alveolar ruptures and show more open alveoli.

To gain a better understanding of the various events occurring during mechanical ventilation, we decided to isolate RNA from the lung tissue of the mice for RNA sequencing. Figure 3A represents the list of genes that were most significantly differentially expressed and then sorted based on highest expression in the 30 mL/kg group and compared to the lower tidal volumes. A clear trend of increased beta value due to increased tidal volume was observed. The more the mechanical strain, the greater the transcriptional response is. When looking up the genes that were most affected, it became clear that a large proportion of them were related to glutathione production, for instance *Slc7a11, Hmox1, Gclc* and *Gclm.* This led us to investigate this path further. In Fig. 3B, we searched for important glutathione associated genes [16] and it became apparent that genes associated with the master regulator of oxidative response (Nrf2) were highly enriched and this trend increased with higher tidal volume in most cases. *Nfe2l2* is the gene that codes for Nrf2. We found little difference in expression of the gene, but increased expression of genes downstream of this transcription factor. This could mean that, although the gene expression of *Nfe2l2* is unchanged, the activity is increased on the protein level. Further analysis of the oxidative response showed that protein levels of Keap1, the regulator of Nrf2 activity, were lower with increased tidal volume. Keap1 has been shown to being degraded through autophagy [19–21]. That is an indication pointing to the stabilization of Nrf2 and thus resulting in the activation of Nrf2 targeted genes. Interestingly, expected inflammatory associated gene set enrichments, such as INFLAMMATORY_RESPONSE; INTERFERON_ALPHA; INTERFERON_GAMMA and IL6_JAK_STAT3_SIGNALING were not observed, although TNFA_SIGNALING_VIA_NFKB and DNA_REPAIR gene sets were enriched (Fig. 5D, F). This was not to the extent anticipated, but indeed with a stepwise increase with increased tidal volume.

Comparing the gene set enrichment scores of enriched gene sets (positively and negatively), showed that important gene sets involved in mitochondrial biogenesis and activity, MYC_TARGETS_V1, MYC_TARGETS_V2 and MTORC1_SIGNALING [24–28] were enriched in a stepwise manner with increasing tidal volume. Gene set OXIDATIVE_PHOSPHORYLATION showed the same trend (Fig. 5A–C). Mitochondrial swelling had been reported previously as result of MV in a mouse model of VILI [29] and we found similar phenotypical changes probably due to increased mitochondrial stress. Figure 6 shows representative images of alveoli type 2 cells at different tidal volume levels. Although the number of mitochondria stayed consistent in the samples, there were significantly larger mitochondria in the ventilated mice.

## Conclusions

In this paper, we report for the first time a look at global RNA expression in a mouse model of VILI and show that oxidative stress response is highly pronounced, and tidal volume/strain associated. Although Nrf2-dependent oxidative stress has been reported [33, 34], we have not seen an extensive transcriptional analysis before. Oxidative stress in lung injury is well documented [35], but its role in VILI has, in our opinion, been under appreciated. The powerful antioxidant, glutathione, has clear cytoprotective roles and its increased expression due to increased tidal volume is suggestive of a strong biological response to the employed stress of the mechanical ventilator. Glutathione levels are known to reduce inflammation and as such has been implicated as a potential protector against severe/inappropriate inflammation [36] and as a molecular target for sepsis patients in critical care [37]. The results presented in this current study support the role of glutathione as a cytoprotective molecule against oxidative stress and should accentuate the urgency to find a potential therapeutic aspect for it.

## Methods

This study was approved by the Icelandic Food and Veterinary Authority (License no. 2007790). Six-week-old C57BL/6 female mice were obtained from Taconic Biosciences and housed under standard conditions at ArcticLAS small animal facilities in Iceland, according to animal welfare regulations. Mice were housed at the facility for three weeks in order to acclimatize and mature. Mice were randomized into groups of six for each treatment condition.

### Mouse model of VILI

Four groups of mice were used in the study, six in each. The mice weighed 16.3–22.3 g and were anesthetized with an intraperitoneal (i.p.) injection of ketamine (80 mg/kg) and xylazine (10 mg/kg). A tracheotomy was performed and an intubation cannula with Y-adapter OD 1.2 MM (Harvard apparatus, VK32 (#73-2844)) was inserted into the trachea and fixed with a suture. One group was then euthanized with a mixture of Euthasol vet/Lidocaine, and lung tissue and BALF harvested to be used as a control group for comparison between spontaneous breathing (SB) against mechanical ventilation. The other three groups were connected to a mechanical ventilator (VentElite small rodent ventilator; 55-7040, Harvard Apparatus) and ventilated with volume-controlled mode with tidal volumes (V_T_) of 10 mL/kg, respiratory rate of 150 breaths/min (minute ventilation of 1500 mL/min); 20 mL/kg, respiratory rate of 120 breaths/min (minute ventilation of 2400 mL/min) and 30 mL/kg, respiratory rate of 80 breaths/min (minute ventilation of 2400 mL/min). PEEP was maintained at 0 cmH_2_O for all groups. Ventilation occurred with atmospheric air with ratio of oxygen at 21%. Mice were ventilated for a maximum of 4 h. During ventilation, mice were supplemented every 20 min (or as necessary according to clinical signs) with a third of the initial dose of ketamine. Heart rate was monitored by inserting platinum needle electrodes subcutaneously into each front leg and left rear leg and connected to a 12-Lead ECG recorder with LabScribe software (Iworx). Body temperature was maintained at around 37.5 °C with a flexible heating pad connected to a homeothermic monitoring system (Harvard apparatus (#55-7020)). After 4 h of ventilation, the mice were euthanized with a mixture of Euthasol vet/Lidocaine, and lung tissue and BALF harvested. BALF was collected from two 0.5 mL 0.9% NaCl saline washes and centrifuged at 2000 rpm for 3 min to collect pellet debris. BALF, free of cellular debris, was divided into three aliquots and stored at − 20 °C for future analyses.

### ELISA

IL-6 cytokine concentrations in BALF samples were measured by ELISA (R&D Systems, DY406) according to the manufacturer’s instructions. In short, plates were coated with diluted anti-IL-6 antibodies overnight at room temperature (RT) and then blocked with blocking solution for 1 h at RT. Standards and samples were then added, incubated, and washed 4 × with washing solution. Detection antibodies diluted in ELISA buffer were then added and incubated for 2 h at RT. Streptavidin-HRP (1:40 in ELISA buffer) was added and incubated in the dark at RT for 20 min. Substrate solution was then added and incubated in the dark at RT until colored precipitates were evident in most concentrated standards. A stop solution was added, and absorbance of the plates were measured on a microplate reader.

### Transmission electron microscopy

Tracheas and lung tissue samples were treated for TEM imaging using standard techniques, previously described in [38], where 100 nm sections were cut with an Ultramicrotome (Leica EM UC7). Sections were stained with lead citrate (3%, J.T. Baker Chemical Co.) and imaged using a JEM-1400PLUS PL Transmission Electron Microscope.

### Protein quantification of BALF samples

Protein concentration of the collected BALF was measured using Bradford reagent (Sigma). A standard curve was prepared using bovine serum albumin, diluted in 0.9% NaCl (same solution as used for BALF collection.) Absorbance values from BALF samples of unknown concentration were fitted to the standard curve for quantification.

### Western blotting

Total protein content was harvested from lung tissue samples by homogenizing the tissue on ice in RIPA buffer (50 mmol/L tris–HCL (pH 7.4), 150 mmol/L NaCl, 0.5% Igepal, 5 mmol/L EDTA (pH 8.0), 0.1% SDS, supplemented with Halt Protease and Phosphatase Inhibitor cocktail (Thermo Fisher). The protein concentration of each sample was determined using Bradford reagent and bovine serum albumin diluted in RIPA. Equal volumes of the samples were then size separated on NuPAGE™ 4–12% Bis–Tris gel (invitrogen by Thermo Fisher Scientific) and transferred to polyvinylidene difluoride transfer membranes (Thermo Scientific). The membranes were blocked in 5% BSA in TBST and probed with primary antibodies (beta actin 20536-1-AP, KEAP1 10503-2-AP, Proteintech) at 4 °C overnight.

Keap1 bands were normalized against actin and quantified using FIJI [39].

### RNA isolation and RNA sequencing

Tissue samples from lung were stored in RNAlater™ (Invitrogen, ThermoFisher) at − 20 °C until total RNA was extracted in TRI-Reagent® (Ambion, ThermoFisher) using M tubes in a gentleMACS™ Dissociator from Miltenyi Biotec. RNA sequencing was performed by BGI Tech Solutions (Hong Kong) Co., Limited, and sequenced with non-stranded mRNA sequencing on 100PE of DNGseq platform. The RNA transcript expression was quantified with Kallisto version 0.46.1 [40] using the Ensembl *Mus musculus* GRCm38 reference transcriptome [41]. Gene expression estimates were computed with the sleuth R package v0.30 [42]. Gene set enrichment analysis was performed with the GSEA software v4.0.1 [22], using the GSEA Hallmark pathway database.

### Image analysis

Lung tissue was fixed, and paraffin embedded, cross-sectioned and hematoxylin and eosin (H&E) stained. Images were captured using an EVOS FL Auto 2 (Invitrogen, ThermoFisher). Tissue histology was assessed using Cell Profiler 3 (Broad Institude, Boston, MA). Briefly, a minimum of 10 images in 10× magnification were acquired from each H&E stained slide of mouse lungs. One slide from each animal in the study was used, for a total of 19 slides and 215 images across the treatment panel. Images were imported into CellProfiler, inverted and channels separated. The red channel was used to identify the number of nuclei, whereas the green channel was used to identify tissue. From those two object groups, within each image, we were able to identify cell number, tissue coverage and open airways, as presented in Fig. 2.

### Statistical analysis

Statistical significance was determined using a student *t*-test and a one-way ANOVA using GraphPad Prism 8.0. Error bars represent the standard deviation of the means. Calculations for the statistical significance for the differential gene expression were calculated using the Wald test in sleuth.

## Supplementary Information

**Additional file 1. Figure S1: **Whole lung sections. Shown are whole lung sections from SB and different tidal volume ventilated mice. The boxes drawn show areas represented in Fig. 3. Width of box is 450 µm and height is 330 µm

**Additional file 2. Figure S2: **Junctional gene sets negatively enriched due to mechanical ventilation. APICAL_JUNCTION gene set is negatively correlated due to mechanical ventilation (A). CLDN4 expression is increased due to mechanical ventilation. Standard deviation of the means is shown. Significant difference from the SB controls are shown (*P* ≤ 0.01 = **; *P* ≤ 0.001 = ***)

## Data Availability

All data is available upon request.

## Abbreviations

ARDS: Acute respiratory distress syndrome
BALF: Bronchoalveolar lavage fluid
COPD: Chronic obstructive pulmonary disease
GSEA: Gene set enrichment analysis
Keap1: Kelch-like ECH-associated protein 1
MV: Mechanical ventilation
NES: Normalized enrichment score
Nrf2: Nuclear factor-erythroid 2-related factor 2
PEEP: Positive end-expiratory pressure
ROS: Reactive oxygen species
VILI: Ventilator-induced lung injury
