# Supplementary figures and images for: Ventilator-induced lung injury results in oxidative stress response and mitochondrial swelling in a mouse model
Source: Lab Anim Res. 2022 Jul 22;38:23. doi: 10.1186/s42826-022-00133-4 (PMC9308307; doi:10.1186/s42826-022-00133-4)

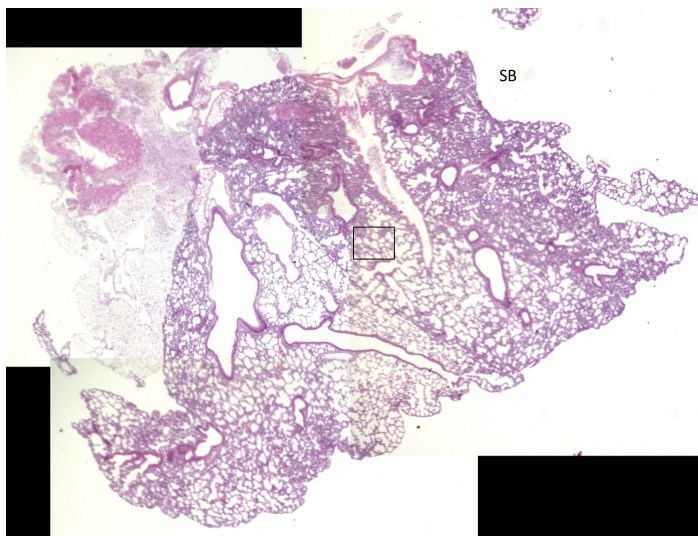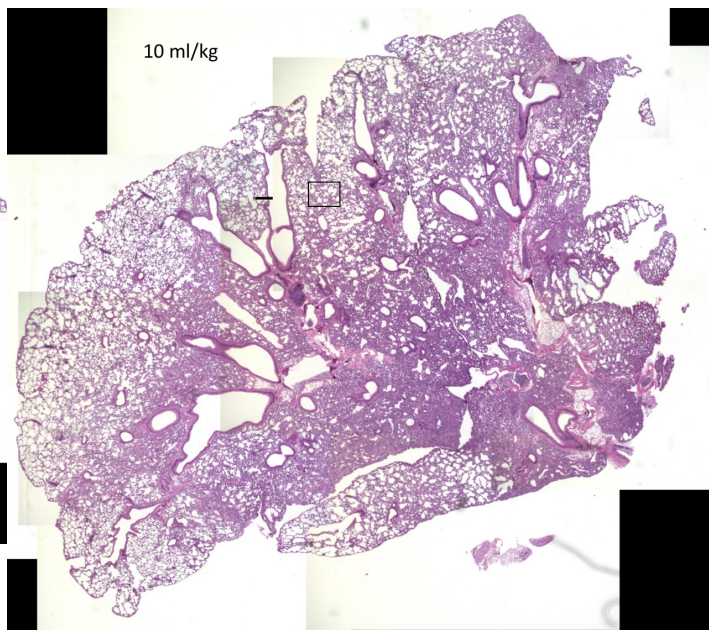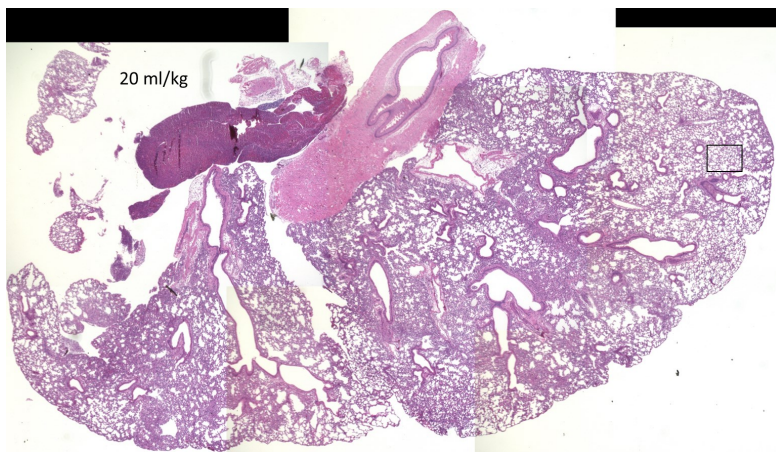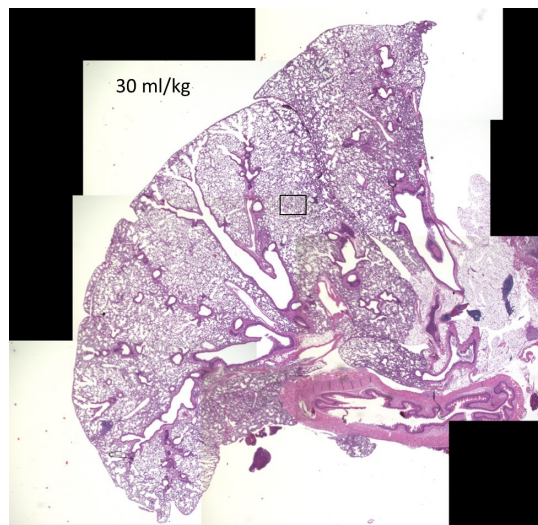

Supplement: Supplementary file 1 — Additional file 1. Figure S1: Whole lung sections. Shown are whole lung sections from SB and different tidal volume ventilated mice. The boxes drawn show areas represented in Fig. 3. Width of box is 450 µm and height is 330 µm [file 42826_2022_133_MOESM1_ESM.pdf]

A

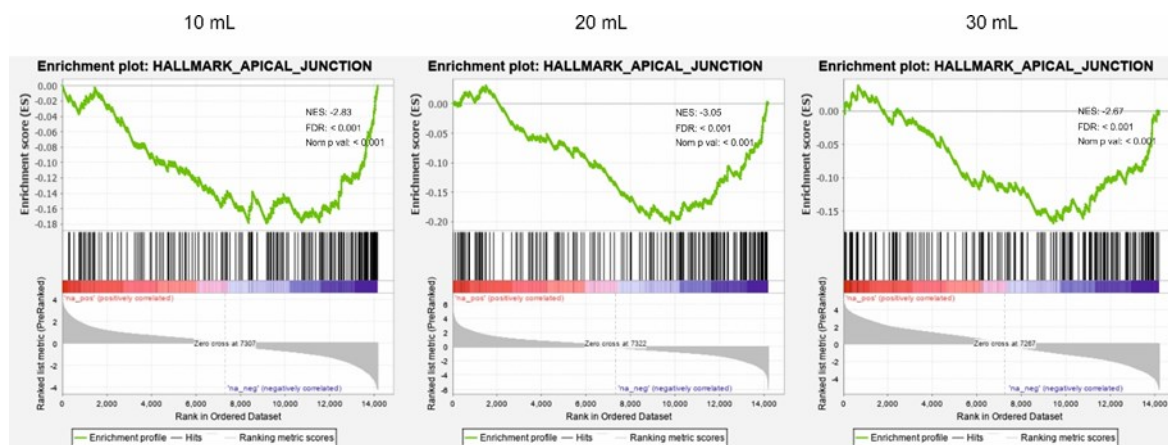

B

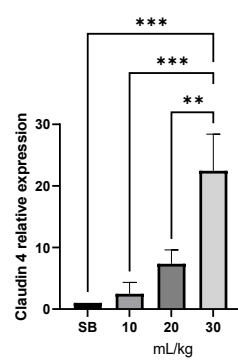

Supplement: Supplementary file 2 — Additional file 2. Figure S2: Junctional gene sets negatively enriched due to mechanical ventilation. APICAL_JUNCTION gene set is negatively correlated due to mechanical ventilation (A). CLDN4 expression is increased due to mechanical ventilation. Standard deviation of the means is shown. Significant difference from the SB controls are shown (P ≤ 0.01 = **; P ≤ 0.001 = ***) [file 42826_2022_133_MOESM2_ESM.pdf]
